# Implementation of enhanced recovery in cardiac surgery: An experimental study with the control group

**DOI:** 10.1177/02184923221138504

**Published:** 2022-11-14

**Authors:** Ayman Hendy, Claudio DiQuinzo, Mark O'Reilly, Abdelaziz Hendy, Michael Vician, Chris Theriault, Edgar Chedrawy, Gregory Hirsch, Hashem Aliter

**Affiliations:** 1 Department of Anesthesia, Pain Management & Perioperative Medicine, 3688 Dalhousie University, Halifax, NS, Canada; 2 Faculty of Medicine, 3688 Dalhousie University, Halifax, NS, Canada; 3 8637 University of Windsor, Ontario, Canada; 4 Research Methods Unit, 432234 Nova Scotia Health Authority, Halifax, NS, Canada; 5 Division of Cardiac Surgery, 3688 Dalhousie University, Halifax, NS, Canada

**Keywords:** Enhanced recovery after surgery, cardiac surgery, perioperative management

## Abstract

**Introduction:**

The Enhanced Recovery After Cardiac Surgery protocol is the most recent addition to cardiac treatment. In this paper, we aimed to test the safety and viability of this protocol in our hospital to improve our standard of care.

**Methods:**

This study was conducted as an experimental study with a historical control at the Maritime Heart Center, Halifax, Nova Scotia, Canada. In order to quantify the success of this protocol, we measured the postoperative Length of Hospital Stay and three intensive care unit variables: time to extubation, time to ambulation, and opioid consumption. In the study, 100 patients were in the Enhanced Recovery After Cardiac Surgery group, and 103 patients were used as historic controls-selected by strenuous chart review and selection criteria.

**Results:**

The primary outcome (Length of Hospital Stay) was reduced from a mean of 8.88 ± 3.50 days in the control group to a mean of 5.13 ± 1.34 days in the Enhanced Recovery After Cardiac Surgery group (*p* < 0.001). Likewise, we observed a significant reduction in intensive care unit variables: time to extubation was reduced from 10.54 ± 7.83 h in the control group to 6.69 ± 1.63 in the Enhanced Recovery After Cardiac Surgery group (*p* < 0.01), and time to ambulation was reduced from 36.27 ± 35.21 h in the control group to 9.78 ± 2.03 in the Enhanced Recovery After Cardiac Surgery group (*p* < 0.01) and opioid consumption was reduced from 50.58 ± 11.93 milligram morphine equivalent in the control group to 11.58 ± 4.43 milligram morphine equivalent in the Enhanced Recovery After Cardiac Surgery group (*p* < 0.01).

**Conclusion:**

Enhanced Recovery After Cardiac Surgery protocols were seamlessly integrated into selected cardiac surgical patients, contingent on a high level of interprofessional communication and collaboration.

## Introduction

Heart disease is the leading cause of death worldwide, accounting for one-third of deaths globally.^1^ Cardiac surgery is used to treat structural and vascular diseases of the heart and is considered the final treatment option in the late stages of these diseases. Consequently, cardiac surgical patients are usually older, sicker, and require a longer hospital stay compared to other surgical patients.^2^

The idea of fast-tracking cardiac surgical patients to minimize intensive care unit (ICU) stay was first developed in the 1990s, however, it did not stand the test of time due to lack of standardized protocol and preoperative patient optimization.^3^

A newer concept, *Enhanced Recovery After Surgery* (ERAS) was first developed for colorectal surgery, and slowly found its way to other surgical specialties including cardiac surgery.^4^ This protocol aims to reduce patient hospital stay through a multidisciplinary approach and standardized perioperative protocols.

The ERAS cardiac protocol was published in 2018 and was based on three main tenets: preoperative, intraoperative, and postoperative interventions.^5^

Cardiac ERAS is different from the classical care pathway in prehabilitation, preoperative oral carbohydrate loading, preferential use of non-opioid analgesia, early postoperative enteral feeding, and early patient mobilization in the ICU.

To date, few studies have been published testing ERAS cardiac applicability and safety.

## Methods

### Study design

Experimental with historic control carried out at the Maritime Heart Center, Queen Elizabeth II Health Sciences, Halifax Infirmary Hospital (Halifax, Nova Scotia, Canada).

The study population was categorized into two groups:

1. ERAS group which consisted of 100 prospective patients that will be having cardiac surgery at Maritime Heart Center in 2019 and 2020.
2. Control group which consisted of 103 retrospective patients (charts) who had similar types of cardiac surgery in 2017 (in the same hospital, by the same group of surgeons).

### Ethics approval

The study was approved by the Research Ethics Board (REB) of Nova Scotia Health (REB file #1023804).

The long-term goal of this study is to make ERAS cardiac protocol the standard of care in elective and urgent cardiac surgery at our hospital.

The stakeholders for this study were cardiac surgeons, anesthesiologists, intensivists, dietitians, physiotherapists, intensive care nurses, patients, and their families.

#### Sample size calculation

The historic estimate of the average length of hospital stay (LOS) after cardiac surgery at our center is 8.88 ± 3.44 days (2017 databases). 2017 was selected because this is the most recent year before any the ERAS interventions was introduced to our patients.

We aimed to reduce LOS by a clinically relevant value of 25% (of 8.88 days **≈** 2 days), expecting the ERAS group to have an average LOS of 6.88 days. Using G* power software to calculate the sample size, it was determined that 128 patients were required in total (64 patients per group). We involved 100 patients per group to compensate for unexpected occurrences.

#### Inclusion criteria for both groups

Elective and urgent cardiac surgery through median sternotomy in patients aged 18–85 years booked for coronary artery bypass grafting (CABG) and/or aortic, mitral, and tricuspid valve surgery with cardiopulmonary bypass (Table 1).

#### Exclusion criteria for both groups

We excluded four types of surgical procedures from the study: off-pump CABG (OPCAB), surgery for atrial septal defect (ASD) repair, left ventricular assist device (LVAD) insertion, and heart transplant.

The first two usually spend the least time in the hospital postoperatively while the latter two require complex care and tend to spend more time postoperatively.

Prospective ERAS patients went through a stepwise screening as follows:

1. All patients completed a questionnaire about their medical history to exclude patients with a history of stroke, previous cardiac surgery, mobility disorders, body mass index > 35, and patients living alone.
2. Investigations of potential patients were checked for hemoglobin level (Hb), kidney function (estimated glomerular filtration rate, eGFR), and cardiac ejection fraction (EF) by echocardiography.
3. Patients were deemed eligible if they had Hb > 125 g/L, eGFR > 60 mL/min/1.73 m^2^, and EF > 35%. These set laboratory values are based on articles showing higher morbidity (prolonged hospital stay after cardiac surgery) and mortality in patients failing to meet these criteria.^6–8^
4. All the 100 eligible patients were approached by a research assistant (RA) holding an MSc degree in Medical Science to explain the project to them. If patients agreed, then one of the surgeons obtained their consent as required in our hospital.
5. Recruited patients were offered educational videos and spoke to the RA about ERAS cardiac before surgery.
6. Postoperatively, patients went through another screening to exclude patients exceeding the recommended safe cardiac ischemic times, that is, aortic cross-clamp > 150 min, or cardiopulmonary bypass time > 240 min. Many clinical studies have shown that the duration of the aortic cross-clamp and CPB times are independent predictors of mortality and morbidity. There is no agreement on safe durations, so we accepted the numbers suggested by Nissinen et al.^9,10^
7. ERAS interventions (preoperative, intraoperative, and postoperative) were done on all subjects of the experimental group (100 patients).

The control group consisted of 103 retrospective patients who already had surgery in our hospital in 2017. All patients who had surgery in 2017 were considered and only those who passed the exclusion criteria were selected.

Tables 1 and 2 show the details of the inclusion and exclusion criteria.

**Table 1.** Enhanced Recovery After Surgery (ERAS) interventions were conducted, pre-, intra-, and post-operatively.

Preoperative interventions	Intraoperative interventions	Postoperative interventions
Preoperative patient engagement and education	Standardized anesthesia technique using short-acting anesthetics by infusion	Non-opioid pain control: 1—IV acetaminophen 2—Serratus anterior plane block (SAPB, Figure 1)
Prehabilitation (pamphlets with simple exercises)	Antibiotics prophylaxis 30–60 min before skin incision	Strict maintenance of normothermia in ICU (36.5 °C–37.2 °C)
Smoking and alcohol session if possible	Two types of antiemetics: early and late-onset nausea and vomiting	Encouraging early tracheal extubation by discontinuing sedation when patients are stable
Preoperative carbohydrate drink (2 h before surgery)	Strict post-cardiopulmonary bypass temperature control (36.5 °C–37.2 °C)	Goal-directed transfusion practice using ROTEM-guided protocols for blood products
Pre-emptive analgesia: acetaminophen (650 mg) and gabapentin (300 mg) 30 min before anesthesia	Standardized antifibrinolytic protocol	Early enteral feeding starting with chewing gum 4 h after tracheal extubation
	Rigid sternal fixation	Early mobilization starting with sitting at the edge of the bed for 4 h after tracheal extubation.

**Table 2.** Inclusion and exclusion criteria for patient enrollment were used for both the experimental and control groups.

Inclusion	Exclusion
Age 18–85 y	Age < 18 y or > 85 y
American Society of Anesthesia ASA classes 2, 3, 4	Patients with overt congestive heart failure (CHF)
Elective or semi-elective cardiac surgery, coronary artery bypass graft (CABG), cardiac valve repair or replacement via median sternotomy, and cardiopulmonary bypass	Emergency cases, for example, aortic dissection Patients on mechanical cardiac support [extracorporeal membrane oxygenator (ECMO) and/or intra-aortic balloon pump (IABP)
	Patients with mobility problems
	Patients who lack support at home
	History of stroke or endocarditis
	Preoperative hemoglobin (Hb) < 125 g/L
	Body mass index (BMI) > 35
	Estimated glomerular filtration rate (eGFR) < 60 mL/min/1.73 m²
	Preoperative ejection fraction (EF) < 35% by echocardiography
	Surgery requires deep hypothermic circulatory arrest (DHCA)
	Percutaneous interventions such as transcatheter aortic valve implantation (TAVI)
	Surgery for a heart transplant
	Surgery for ventricular assist device insertion
	Abnormal liver function tests: albumin < 30 g/L, AST, ALT > double normal range for both (i.e. > 100 units/L)
	Intraoperative variables excluded from the analysis
	Aortic cross-clamp (AOX) > 150 min
	Cardiopulmonary bypass time (CPB) > 240 min
	Inability to close the chest due to (bleeding, hemodynamic instability)
	Postoperative need for IABP

**Figure 1.**
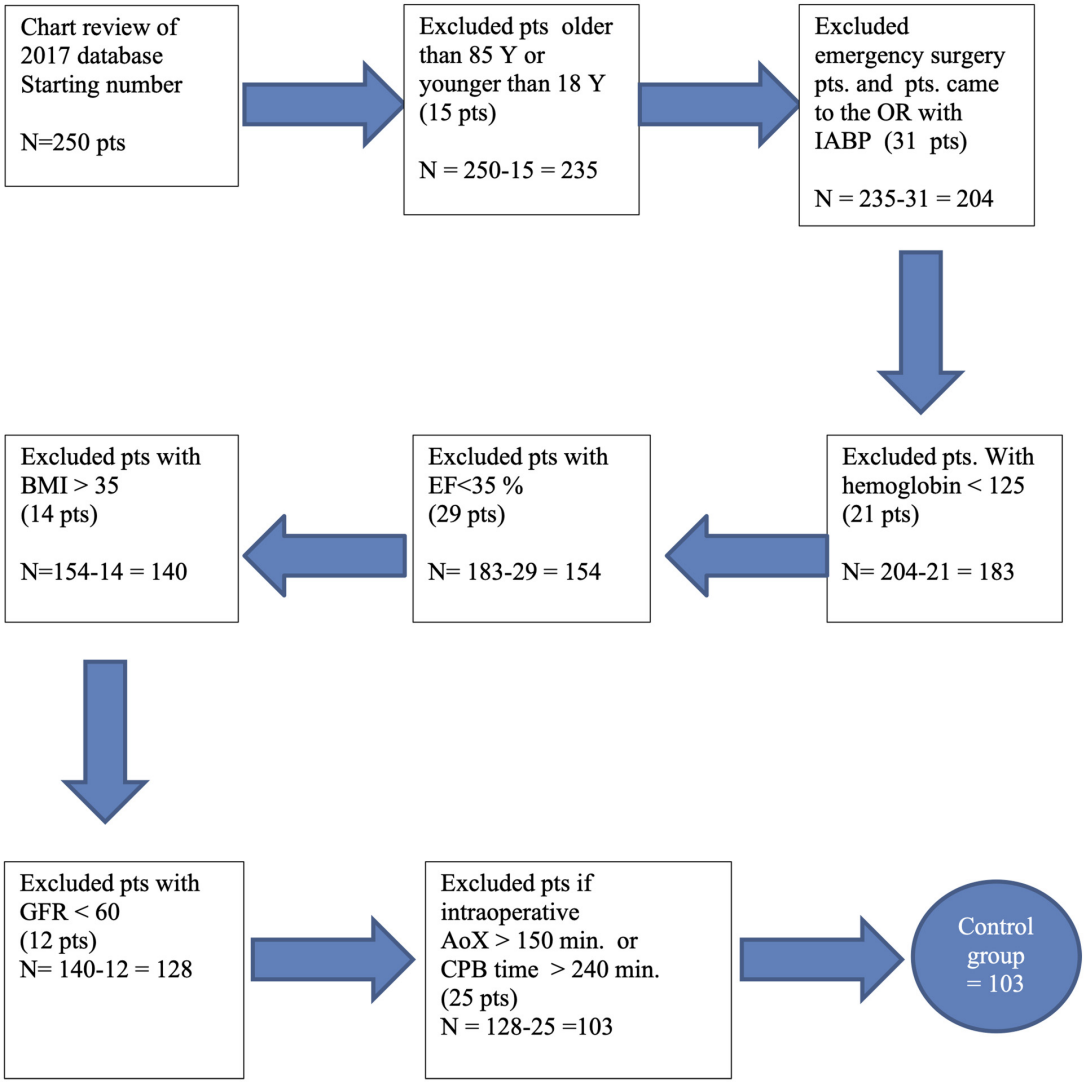
Flowchart demonstrating the selection process of the matched control group.

Figure 1 shows how the control group was selected.

#### ERAS preoperative care

A few days prior to surgery, patient education and prehabilitation (encouraging healthy eating and simple exercises) were ensured by phone calls for patients coming from a home or by nursing staff for inpatients.

On the day of surgery, ERAS patients were given a carbohydrate load consisting of 200 mL of apple juice (24 g of glucose) 2–3 h before surgery to maintain gut motility and to be used as an energy source (protein-sparing effect).^11^

Oral analgesia (650 mg of Acetaminophen and 300 mg of Gabapentin) was administered 30 min before anesthesia induction.

These four measures are the core differences between cardiac ERAS and the old fast-tracking concept popular in the 1990s.

### Intraoperative care

The routine intraoperative care of elective cardiac surgical patients at the Halifax Infirmary hospital occurs in the following sequence: the patient arrives at the operating room on a stretcher and moves to the operating table where monitors are applied, that is, five lead ECG, noninvasive blood pressure, pulse oximeter, bispectral index, and near-infrared spectroscopy (NIRS).

After the above monitors are applied, a large bore intravenous is inserted and 1–2 mg of IV Midazolam is given before starting the invasive arterial line.

Surgical timeout is led by the anesthesiologist in the presence of all members of the operating team.

Induction of anesthesia starts after 3 min of preoxygenation followed by IV injection of sufentanil (0.5–1 mcg/kg), ketamine (0.5 mg/kg), propofol (1–2 mg/kg), and rocuronium (1.5 mg/kg) sequentially.

Endotracheal intubation follows induction and treatment of any hemodynamic instability occurs simultaneously. Next, central venous line insertion is performed under a strict aseptic technique followed by urinary catheterization.

IV antibiotics (2 g cefazolin), or 1 g vancomycin if allergic to cephalosporin) are administered after completion of the central line and within 30–60 min before skin incision. Maintenance of anesthesia in the pre-bypass period is done using a continuous infusion of sufentanil 0.2–0.6 mcg/kg/h and sevoflurane anesthetic gas (1.5–2.5%) to achieve a minimum alveolar concentration (MAC) of 0.8.

During bypass time, Sevoflurane is turned off and propofol is infused at 80–150 mcg/kg/min instead. Sufentanil infusion (0.2–0.6 mcg/kg/h) continues until the end of surgery.

Weaning from bypass is done 10 min after cross-clamp removal with stable heart rate, contractility, and hemodynamics. Pacing and inotropes are used as needed. The sternum is closed when hemostasis is satisfactory.

### Postoperative care

Patients are transferred to the ICU intubated, ventilated, sedated, and monitored.

On arrival to the ICU, patients get connected to the ventilator and a report is given to ICU staff after ensuring hemodynamic stability.

The routine postoperative care in the ICU consists of the following:

1. Cardiac monitoring: vitals monitored every 15 min, four times, then every 30 min four times, then every hour, and as required.
2. ECG and chest X-ray 2 h post-admission.
3. Chest tubes are connected to underwater drainage with 15 cmH_2_O suction. The surgeon is contacted if tube drainage is > 150 mL in the first 2 h.

Weaning from ventilatory support is considered after 2 h of stable hemodynamics and minimal chest tube drainage. It is performed with an intensivist and the respiratory therapist guidance and starts with cessation of propofol sedation to allow for spontaneous breathing. The ventilator setting is then switched to pressure support of 6 cmH_2_O and CPAP of 5 cmH_2_O. Extubation follows when tidal volume is > 5 mL/kg, respiratory rate < 25/min, vital capacity > 10 mL/kg, and PaO_2_/FIO_2_ > 200.

#### Pain management (control group, standardized analgesia protocol)

From the time of arrival to the ICU until extubation, fentanyl is infused at 25–100 mcg/h. After extubation, fentanyl 25 mcg is delivered directly through IV injections every 5 min, as needed (with a maximum of 300 mcg over 6 h). Hydromorphone (1–2 mg) is delivered subcutaneously every 3 h as needed, and 2–4 mg orally every 3 h as needed.

#### Non-opioid adjuvants

Acetaminophen 1000 mg every 6 h, not to exceed 4 g/day.

Ketorolac (15 mg) is given intravenously every 8 h (not given if bleeding or if the patient has abnormal kidney function).

#### Pain management (ERAS group)

Ultrasound-guided serratus anterior plane block is performed within one hour from arrival to ICU.

Fentanyl and acetaminophen in a similar dose and regimen as described above are made available to patients if the nerve block was not sufficient.

#### Delirium assessment (both groups)

Richmond Agitation Sedation Score (RASS) and Confusion Assessment Method (CAM ICU) are checked every 4 h after extubation.

#### Discharge from ICU to step-down unit (both groups)

To be eligible for discharge from the ICU patients must meet the following criteria:

1. If pace, they must have an underlying rhythm.
2. No intermittent positive pressure ventilation (can be on O_2_ nasal cannula).
3. Minimal hemodynamic support (i.e. epinephrine ≤ 0.05 mcg/kg/min).
4. Oral feeds.
5. No progressive renal failure.
6. No hyperactive delirium.

#### Statistical analysis

Descriptive statistics are expressed as mean ± standard deviation for symmetrically distributed variables; median and interquartile ranges for skewed variables; and percentages for categorical variables. We compared extubation time (cessation of mechanical ventilation in ICU), opioid consumption in ICU, rate of complications, and LOS before and after the implementation of the ERACS protocol.

All analyses were performed with STATA software version 14.2. The difference was considered statistically significant if the resulting *p*-value was < 0.05.

Normality tests (Shapiro-Wilk) showed that all numerical outcome variables deviate from a normal distribution, therefore, non-parametric tests (Mann-Whitney) were used to compare groups. Categorical variables were compared using Chi-squared and Fisher's exact tests. The adjusted analysis was performed using quantile regression (median regression) with three predictors: group, age, and sex.

#### Results

The control group consisted of 103 patients who underwent cardiac surgery at the Halifax Infirmary hospital in 2017 (the total number of cardiac surgeries in the hospital that year was 903). Since randomization of the study and control groups was not feasible, propensity score matching was used. Both groups underwent the same screening process and were matched to the nearest neighbor matching (Table 3).

**Table 3.** Preliminary analysis to ensure matching of ERACS and control groups.

Demographics and medical characteristics	Control group *N* = 103	ERACS group *N* = 100	Comparison between groups
Age mean ± SD [range]	64.08 ± 9.70 [41–85]	63.07 ± 10.70 [34–85]	*t*(201) = 2.10, *p* = 0.037
Patient gender, *n* (%) Male Female	69 (67%) 34 (33%)	82 (82%) 18 (18%)	χ²(1) = 5.32, *p* = 0.021
Body mass index (BMI), *M* ± *SD* [range]	30.24 ± 5.79 [18.87–52.44]	29.50 ± 5.61 [19.62–50.07]	*t*(201) = 0.92 *p* = 0.36
Comorbidities (*n* and %)			
Hypertension	76 (74%)	64 (64%)	χ²(1) = 2.27, *p* = 0.13
Diabetes	31 (30%)	27 (27%)	χ²(1) = 0.24, *p* = 0.63
Peripheral vascular disease (PVD)	9 (9%)	2 (2%)	Fisher's exact *p* = 0.06
Chronic obstructive pulmonary disease (COPD)	15 (15%)	12 (12%)	χ²(1) = 0.29, *p* = 0.59
Atrial fibrillation (AF)	8 (8%)	5 (5%)	χ²(1) = 0.65, *p* = 0.42
Type of surgery (*n* and %)			
Aortic valve replacement (AVR)	26 (25%)	26 (26%)	
Coronary artery bypass graft (CABG)	69 (67%)	56 (56%)	
AVR + CABG	3	4 (4%)	
Mitral valve surgery (MVR)	3	5 (5%)	
Tricuspid valve surgery (TVR)	1	5 (5%)	
Others	1	4 (4%)	
Cardiopulmonary bypass (CPB) time (min)	106.15 ± 32.76 [41–207] Med = 101 (87–121)	107.86 ± 33.81 [37–194] Med = 104 (80–128)	M-W *p* = 0.79 ANCOVA *p* = 0.92 QR *p* = 0.52
Aortic cross-clamp time (AOX)	80.94 ± 28.35 [29–150] Med = 78(65–91)	82.19 ± 28.11 [20–150] Med = 81 (60–90)	M-W *p* = 0.54 ANCOVA *p* = 0.91 QR = .82

*Note:* No statistical analysis was conducted to compare surgery types between the groups.

The primary outcome observed was the length of postoperative hospital stay, which was reduced from a mean of 8.88 ± 3.50 days in the control group to a mean of 5.13 ± 1.34 days in the ERAS group. All patients (ERAS and control) were discharged home. Patients with mobility limitations or living alone were not engaged in the ERAS group and were matched in the control group.

Secondary outcomes observed were time to extubation, time to ambulate, opioid consumption, and rate of complications in both groups. Among the control and intervention groups, the time to extubate was reduced from 10.54 ± 7.83 h to 6.69 ± 1.63 h and the time to ambulate was reduced from 36.27 ± 35.21 h to 9.78 ± 2.03 h. Opioid consumption was reduced from 50.58 ± 11.93 milligram morphine equivalent (MMI) to 11.58 ± 4.43 MMI. Unadjusted and adjusted analysis results were consistent, suggesting a significant improvement in all outcomes for the ERAS group.

The rate of post-operative complications while in hospital showed a general downward trend post-ERAS implementation, however, results were comparable in both groups and did not appear to be statistically significant. Additionally, no in-hospital mortality or 30 days (all causes) mortality was observed in either control or intervention group in the study.

Of special importance in the complications recorded was post-operative kidney dysfunction, defined as a 50% increase in serum creatinine from the baseline level and progressive decline in urine output.^12^ Usually this is seen in up to 30% of cardiac surgical patients postoperatively.^13,14^ We recorded a transient increase in the measured creatinine level, but none of our patients required dialysis. This may be due to the inclusion of patients with adequate kidney function (i.e. GFR > 60) only. All patients were discharged home, as our exclusion criteria included patients with mobility problems and those without support at home.

Tables 4 and 5 summarize the results and the complications recorded.

**Table 4.** Comparison of the primary and secondary outcomes between control and ERAS groups.

Primary and secondary outcomes	Control group *n* = 103	ERACS (intervention group) *n* = 100	Comparison between groups
Time to extubation (hours)	14.72 ± 33.15 [2–313] Med = 9 (5–13)	6.69 ± 1.64 [4–15] Med = 6 (6–8)	M-W *p* < 0.001 ANCOVA *p* = 0.022 QR *p* = 0.003
Hospital length of stay (days).	8.88 ± 3.50 [4–25] Med = 8 (7–9)	5.13 ± 1.34 [3–11] Med = 5 (4–6)	M-W ***p*** < 0.001 ANCOVA ***p*** < 0.022 QR ***p*** < 0.003
95% confidence interval estimated using bootstrapping method	78.26–9.60	4.95–5.05	
Time to ambulate (Sitting at the edge of the bed) (hours)	40.43 ± 46.70 [13–313] Med = 23 (21–40)	9.78 ± 2.03 [7–20] Med = 10 (8–11)	M-W ***p*** < 0.001 ANCOVA ***p*** < 0.001 QR ***p*** < 0.001
95% confidence interval estimated using bootstrapping method	22.02–23.98	9.16–10.84	
Opioid consumption in ICU milligram morphine equivalent (MMI)	50.58 ± 11.93 [30–100] Med = 50 (40–55)	11.58 ± 4.43 [5–25] Med = 10 (10–15)	M-W ***p*** < 0.001 ANCOVA ***p*** < 0.001 QR ***p*** < 0.001

**Table 5.** Postoperative complications.

Complication	Control group *n* = 103	ERAS (intervention group) *n* = 100	Comparison between groups
Atrial Fibrillation	15 (14%)	13 (13%)	Fisher's exact *p* = 1.00
Urinary tract infection	6 (6%)	6 (6%)	Fisher's exact *p* = 1.00
Pneumonia	2 (2%)	0 (0%)	Fisher's exact *p* = 0.50
Wound infection	12 (12%)	4 (4%)	Fisher's exact *p* = 0.07
Stroke	1 (1%)	0 (0%)	Fisher's exact *p* = 1.00
Readmission to ICU	10 (10%)	8 (8%)	Fisher's exact *p* = 0.81
Acute kidney injury requiring dialysis	0	0	
In hospital mortality	0	0	
30 days (all causes) mortality	0	0	

*Note:* Reported values are *M* ± *SD* [range], median (IQR); IQR = inter-quartile range.

M-W = Mann-Whitney test (the non-parametric equivalent of independent samples *t*-test).

QR = quantile regression; ICU: intensive care unit; ERAS: Enhanced Recovery After Surgery.

## Discussion

Though ERAS has already been proven effective in several noncardiac surgeries including colon and lung surgeries,^15–17^ cardiac surgery has its unique challenges due to the inherent characteristics of cardiac surgical patients and the major derangement of the patients’ internal homeostasis caused by the cardiopulmonary bypass circuit.^2,18^ Spanning the ERAS interventions over the perioperative period seemed to add marginal gains leading to the success of the protocol.^19^ Prior to the development of the ERAS protocol in 2018, a randomized clinical trial by Li et al.,^20^ applied similar interventions to patients undergoing elective cardiac valve replacement surgery. Here, paravertebral nerve blocks to decrease opioid consumption were used and erythropoietin (EPO) injections were administered to their patients to increase red cell mass and avoid blood transfusion. In our study, a Serratus Anterior Plane Nerve Block (SAPB) was used to avoid potential spinal hematoma in patients receiving intraoperative anticoagulation, and we showed a similar reduction in opioid use. Additionally, the ERAS protocol does not include EPO injections, and as such, it was excluded from our study. Instead, we excluded patients with preoperative hemoglobin < 125 g/L, based on previous literature.^6^

Despite differences in study designs, our study was in accordance with the 2020 study by Grant et al.,^21,22^ where strict adherence to ERAS interventions was evaluated in conjunction with three outcomes: time to extubation, LOS, and opioid consumption.

In summary, we believe that the following five key interventions were responsible for the success of ERAS cardiac in our hospital:

- **Patient education in the preoperative period**, which allowed realistic expectations from patients, and it has been an indispensable step in the success of many ERAS programs.^23^ In our case, patient education involved three measures: patients spoke with an experienced RA to answer their questions and alleviate their fears, were provided with reading materials with illustrations including exercises, and finally, our hospital made several YouTube videos about ERAS cardiac available to patients and their families.
- **The modification of fasting guidelines**, allowing patients to drink 200 mL (24 g of glucose) of commercially available apple juice 2 h before surgery, in opposition to the common preoperative fasting guidelines published by the American Society of Anesthesiologists over two decades ago.^24^ The implementation of consuming clear carbohydrates such as apple juice 2 h before surgery has shown to be beneficial in preventing protein catabolism, leading to its recent popularity.^25^
- **Pre-emptive analgesia**, where the analgesic regimen is introduced prior to the onset of noxious stimuli to aid in preventing central nervous system sensitization caused by the incision. This concept was first introduced by Crile, who recommended using local anesthetics through regional nerve blocks in addition to general anesthesia for better control of surgical pain.^26,27^ Our ERAS cohort received acetaminophen and gabapentin orally 30 min before surgery, as both medications have been proven safe in cardiac settings.^28^ Postoperatively, our ERAS patients received an ultrasound-guided SAPB. The maximum dose of local anesthetic used in the ERAS group was calculated based on patient weight and was divided into two doses, given on either side of the chest wall. Additional pain medications were given in the ICU if needed.
- **Early enteral feeding**, a paradigm shift in our study. The first oral intake in our ERAS group was sham feeding (chewing gum) 4 h after extubation (with consideration of the patient condition). This is studied extensively in ERAS protocols and found to be beneficial in accelerating the return of gut motility through neuronal and hormonal effects.^29^ Following this, the ERAS patients were fed semisolid fruit jelly on postoperative day 1 (POD1). No ileus was recorded in our ERAS patients following this protocol.
- **Encouraging early postoperative mobilization**, based on Yayla's protocol of early mobilization after cardiac surgery.^30^ This consists of instructing the patients to use the incentive spirometer, deep breathing, and coughing exercises on POD0 (the day of surgery, 6 h after extubation). Additionally, passive range of motion (ROM) of upper and lower extremities was conducted and patients were encouraged to sit on the edge of the bed, or the bed was inclined to give a sitting position for 15 min twice a day. On POD1, in addition to POD0 protocols, patients were instructed to sit in a chair for 20 min three times a day, and if the patient's condition permitted, they were encouraged to walk 100 steps in the ICU twice daily. POD2-4 patients had usually left the ICU, where the frequency of the antecedent interventions was increased gradually to prepare patients for discharge.

### Study limitations

Like all experimental and observational studies which lack randomization, the internal validity of the study could have been affected. We were aware of this problem, and we exhibited a high level of caution in matching ERAS and control patients.

Additionally, there were no further changes outside the ERAS protocol made to the standard of care after 2017, aiming to eliminate the biases that may result due to the loss of blinding in the intervention groups.

Another limitation of our study was the degree of selectivity in patient recruitment. This can be explained by the high degree of engagement of medical staff in a new protocol which made accepting all prospective cardiac surgery patients a very resource-consuming task. We are sure this will change slowly over time and ERAS will be applicable to all patients in the future.

Furthermore, the COVID-19 pandemic led to a reduced patient recruitment in the later stages of the study, however, the populations of both groups exceeded the calculated sample size.

## Conclusion

Altogether, the successful implementation of the ERAS protocol requires a paradigm shift in cardiac surgery and a great degree of interdisciplinary collaboration—which may not be an easy task for pioneers of cardiac ERAS in their hospital setting.

Future steps include recruiting additional centers to apply ERAS guidelines in cardiac surgery, leading to increased data accumulation to hopefully support ERAS as the new standard of care in elective cardiac surgery. It is also important to note that the very nature of cardiac surgery leads to an extensive patient selection protocol prior to the implementation of ERAS in fear of complications and should be assessed on an individual basis despite its known benefits.

## References

[1] World Health Organization. Cardiovascular diseases (CVDs), https://www.who.int/news-room/fact-sheets/detail/cardiovascular-diseases-2019).

[2] Cornwell LD Omer S Rosengart T, et al. Changes over time in risk profiles of patients who undergo coronary artery bypass graft surgery: the veterans affairs surgical quality improvement program (VASQIP). JAMA Surg 2015; 150: 308–315. 25671647 10.1001/jamasurg.2014.1700

[3] Cheng DC. Pro: early extubation after cardiac surgery decreases intensive care unit stay and cost J Cardiothorac Vasc Anesth 1995; 9: 460–464. 7579120 10.1016/s1053-0770(05)80105-3

[4] Ljungqvist O Scott M Fearon KC. Enhanced recovery after surgery: a review. JAMA Surg 2017; 152: 292–298. 28097305 10.1001/jamasurg.2016.4952

[5] Engelman DT Ali WB Williams JB, et al. Guidelines for perioperative care in cardiac surgery: enhanced recovery after surgery society recommendations. JAMA Surg 2019; 154: 755–766. 31054241 10.1001/jamasurg.2019.1153

[6] Karkouti K Wijeysundera DN Beattie WS. Risk associated with preoperative anemia in cardiac surgery: a multicenter cohort study. Circulation 2008; 117: 478–484. 18172032 10.1161/CIRCULATIONAHA.107.718353

[7] Kolli H Rajagopalam S Patel N, et al. Mild acute kidney injury is associated with increased mortality after cardiac surgery in patients with eGFR < 60 ml/min/1.73 m^2^. Ren Fail 2010; 32: 1066–1072. 20863211 10.3109/0886022X.2010.510616

[8] Li E Nashef SA Roques F, et al. EuroSCORE II. Eur J Cardiothoracic Surg 2012; 41: 734–745. 10.1093/ejcts/ezs043 22378855

[9] Nissinen J Biancari F Wistbacka JO, et al. Safe time limits of aortic cross-clamping and cardiopulmonary bypass in adult cardiac surgery. Perfusion 2009; 24: 297–305. 20007817 10.1177/0267659109354656

[10] Salsano A Giacobbe DR Sportelli E, et al. Aortic cross-clamp time and cardiopulmonary bypass time: prognostic implications in patients operated on for infective endocarditis. Interact Cardiovasc Thorac Surg 2018; 27: 328–335. 29579243 10.1093/icvts/ivy085

[11] Bilku DK Dennison AR Hall TC, et al. Role of preoperative carbohydrate loading: a systematic review. Ann R Coll Surg Engl 2014; 96: 15–22. 24417824 10.1308/003588414X13824511650614 PMC5137663

[12] O'Neal JB Shaw AD Billings FT IV. Acute kidney injury following cardiac surgery: current understanding and future directions. Crit Care 2016; 20: 187–195. 27373799 10.1186/s13054-016-1352-z PMC4931708

[13] Elghoneimy YA Qahtani AA Almontasheri SA, et al. Renal impairment after cardiac surgery: risk factors, outcome and cost effectiveness. Cureus 2020; 12: e11694. 33262922 10.7759/cureus.11694 PMC7689806

[14] Levey AS Eckardt KU Tsukamoto Y, et al. Definition and classification of chronic kidney disease: a position statement from kidney disease: improving global outcomes (KDIGO). Kidney Int 2005; 67: 2089–2100. 15882252 10.1111/j.1523-1755.2005.00365.x

[15] Kehlet H. Fast-track colonic surgery: status and perspectives. Recent Results Cancer Res 2005; 165: 8–13. 15865015 10.1007/3-540-27449-9_2

[16] Batchelor TJ Ljungqvist O. A surgical perspective of ERAS guidelines in thoracic surgery. Curr Opin Anesthesiol 2019; 32: 17–22. 10.1097/ACO.0000000000000685 30589662

[17] Hubert J Bourdages-Pageau E Garneau CA, et al. Enhanced recovery pathways in thoracic surgery: the Quebec experience. J Thorac Dis 2018; 10: 583–590. 10.21037/jtd.2018.01.156 PMC5880988 29629206

[18] Murphy GJ Angelini GD. Side effects of cardiopulmonary bypass: what is the reality? J Card Surg 2004; 19: 481–488. 15548178 10.1111/j.0886-0440.2004.04101.x

[19] Fleming IO Garratt C Guha R, et al. Aggregation of marginal gains in cardiac surgery: feasibility of a perioperative care bundle for enhanced recovery in cardiac surgical patients. J Cardiothorac Vasc Anesth 2016; 30: 665–670. 27321791 10.1053/j.jvca.2016.01.017

[20] Li M Zhang J Gan TJ, et al. Enhanced recovery after surgery pathway for patients undergoing cardiac surgery: a randomized clinical trial. Eur J Cardiothorac Surg 2018; 54: 491–497. 29514224 10.1093/ejcts/ezy100

[21] Grant MC Isada T Ruzankin P, et al. Results from an enhanced recovery program for cardiac surgery. J Thorac Cardiovasc Surg 2020; 159: 1393–1402. 31279510 10.1016/j.jtcvs.2019.05.035

[22] Grant MC Isada T Ruzankin P, et al. Opioid-sparing cardiac anesthesia: secondary analysis of an enhanced recovery program for cardiac surgery. Anesth Analg 2020; 131: 1852–1861. 32889848 10.1213/ANE.0000000000005152

[23] Stenberg U Vågan A Flink M, et al. Health economic evaluations of patient education interventions a scoping review of the literature. Patient Educ Couns 2018; 101: 1006–1035. 29402571 10.1016/j.pec.2018.01.006

[24] Warner MA Caplan RA Epstein BS, et al. Practice guidelines for preoperative fasting and the use of pharmacologic agents to reduce the risk of pulmonary aspiration: application to healthy patients undergoing elective procedures: a report by the American society of anesthesiologists task force on preoperative fasting. Anesthesiology 1999; 90: 896–905. 10078693 10.1097/00000542-199903000-00034

[25] Fawcett WJ Ljungqvist O. Starvation, carbohydrate loading, and outcome after major surgery. BJA Educ 2017; 17: 312–316.

[26] Dahl JB Møiniche S. Pre-emptive analgesia. Br Med Bull 2005; 71: 13–27. 10.1093/bmb/ldh030 15596866

[27] Kaufman E Epstein JB Gorsky M, et al. Preemptive analgesia and local anesthesia as a supplement to general anesthesia: a review. Anesth Prog 2005; 52: 29–38. 15859447 10.2344/0003-3006(2005)52[29:PAALAA]2.0.CO;2 PMC2526217

[28] Maitra S Baidya DK Bhattacharjee S, et al. Perioperative gabapentin and pregabalin in cardiac surgery: a systematic review and meta-analysis. Rev Bras Anestesiol 2017; 67: 294–304. 28258733 10.1016/j.bjan.2016.07.005

[29] Noble EJ Harris R Hosie KB, et al. Gum chewing reduces postoperative ileus? A systematic review and meta-analysis. Int J Surg 2009; 7: 100–105. 19261555 10.1016/j.ijsu.2009.01.006

[30] Yayla A Özer N. Effects of early mobilization protocol performed after cardiac surgery on patient care outcomes. Int J Nurs Pract 2019; 25: e12784. 31617651 10.1111/ijn.12784

